# A comparison of the nutritional profile and nutrient density of commercially available plant-based and dairy yogurts in the United States

**DOI:** 10.3389/fnut.2023.1195045

**Published:** 2023-05-25

**Authors:** Astrid E. D’Andrea, Amanda J. Kinchla, Alissa A. Nolden

**Affiliations:** Department of Food Science, University of Massachusetts Amherst, Amherst, MA, United States

**Keywords:** nutrient composition, nutritional profile, fortification, ingredients, micronutrients, protein, plant-based yogurt, macronutrients

## Abstract

**Introduction:**

Plant-based yogurts are sustainable alternatives to dairy yogurts, but a nutritional comparison of plant-based yogurts within the context of dairy yogurts has not yet been applied to commercially available products in the United States. Dairy yogurts provide significant dietary nutrients, and substituting plant-based yogurts may have unintended nutritional consequences. The objective of this study was to compare the macronutrient and micronutrient values of commercially available plant-based and dairy yogurts launched between 2016 and 2021.

**Methods:**

Nutritional information for yogurts were collected through Mintel Global New Products Database, and products were categorized according to their primary ingredient. Regular-style yogurts (*n* = 612) were included in this study: full-fat dairy (*n* = 159), low and nonfat dairy (*n* = 303), coconut (*n* = 61), almond (*n* = 44), cashew (*n* = 30), and oat (*n* = 15). We utilized the Nutrient Rich Foods (NRF) Index, a comprehensive food guidance system that assigns a score based on the nutrient density of individual foods. This allowed us to compare the nutritional density of the yogurts based on nutrients to encourage (protein, fiber, calcium, iron, potassium, vitamin D) and nutrients to limit (saturated fat, total sugar, sodium).

**Results:**

Compared to dairy yogurts, plant-based yogurts contained significantly less total sugar, less sodium, and more fiber. However, plant-based yogurts contained significantly less protein, calcium, and potassium than dairy yogurts. The yogurts were ranked from the highest to lowest nutrient density based on the NRF Index as follows: almond, oat, low and nonfat dairy, full-fat dairy, cashew, and coconut. Almond yogurts scored significantly higher than all other yogurts, indicating the highest nutrient density.

**Discussion:**

The highest NRF scores were awarded to almond and oat yogurts, likely a result of their low levels of total sugar, sodium, and saturated fat. By applying the NRF model to plant-based and dairy yogurts, we have identified opportunities for the food industry to improve the formulation and nutritional composition of plant-based yogurts. In particular, fortification is an opportunity to improve plant-based yogurt nutritional properties.

## 1. Introduction

The world population is rising and is estimated to reach 9.7 billion by 2050 (1). To sustain this growth, the global food supply chain will require a substantial increase in energy and resources (2). However, these resources are impacted by climate change. The effects of climate change are particularly threatening to the livestock industry: rising temperatures can induce heat stress and thus lower productivity and fertility; emerging vector-borne diseases threaten the population; and climate variability affects the availability of crops for feed (3). In addition to the potential uncertainty of available resources due to climate change, the sustainability of the agricultural food supply must be improved (4). The dairy industry is one critical food production sector with a considerable impact on the environment due to greenhouse gas emissions, use of water resources, and large land requirements (5). For example, the global dairy industry was estimated to have emitted 1,711.8 million tons of CO₂ equivalent in 2015, primarily from enteric fermentation and emissions from feed production and manure management (3). European dairy farms have been estimated to contribute to approximately 80% of the total carbon footprint of dairy products (6). Livestock is directly responsible for 38 and 4% of the United States CH_4_ and NO_2_ emissions, respectively (7). Despite the dairy industry’s effect on the environment, global cow milk production has grown approximately 2.8% per year between 2005 and 2015 (3).

In the U.S., approximately 6.3% of all dairy consumed is in the form of yogurt (8). In 2021, consumers spent $9,246 million on yogurts and yogurt drinks (9). In comparison, the plant-based yogurt market is valued at roughly $1,600 million and is expected to grow to more than $6,500 million by 2030 (10). Within this category, plant-based yogurts have been positioned as a more sustainable alternative to dairy yogurt. Further, plant-based yogurt production emits fewer greenhouse gases and requires less land than the production of dairy yogurts (11, 12). Increased environmental consciousness has been identified as a driver for adopting a plant-based diet (13). However, despite the appeal of the environmental benefits of a plant-based diet, this may not translate to consumer motivation to purchase specific products, like plant-based yogurt. A Mintel report (9) found that among American yogurt buyers, fewer than one-half (42%) perceived plant-based yogurt as more environmentally friendly than dairy yogurt. However, “trying to eat healthier” was the primary motivation for anticipated increased household yogurt consumption (9). Therefore, it has been recommended that plant-based yogurt brands specify the health benefits of their products’ ingredients to attract consumers (9). This presents a challenge: dairy yogurt is considered a nutrient-rich food that offers high quantities of desirable nutrients and, dependent on the type, relatively low amounts of fat and sugar (14). When developing plant-based yogurts, it is important to consider the overall nutritional profile since consumers may utilize these products as a direct substitute for dairy products.

Previous studies have examined the nutritional properties of commercially available plant-based yogurts in the United Kingdom (15), western United States (16), European Union (17), Norway (18), Greece (19), Ireland (20), and across multiple countries (21). Some plant-based yogurt’s nutritional values were found to be significantly different from dairy yogurt’s nutritional values. Significantly different macronutrients have included energy (15, 17), fat (15, 17), saturated fat (15, 19), carbohydrates (15, 17, 19), total sugar (15, 19), fiber (15, 18, 19), protein (15, 17–19), and sodium (15, 18, 19). Additionally, some macronutrients within the plant-based yogurt category were found to be significantly different from each other (16, 19). Regarding micronutrients, calcium levels in plant-based yogurts significantly differed from dairy yogurts (15).

It is important to note that only one plant-based yogurt nutrition study examined products from the U.S. market (16). While Craig and Brothers (16) provided important new insights into the comparison within the plant-based yogurt category, there was no formal comparison to dairy yogurt. The present study is the first to compare the nutritional properties of dairy and plant-based yogurts from the U.S. market. Unlike previous studies examining plant-based and dairy yogurts, here we differentiate between the fat levels of the yogurts, whereas previous studies combined full-fat, low-fat, and nonfat yogurt. This separation provides insight into comparing fat and saturated fat levels in dairy yogurts to plant-based yogurts. Additionally, we conducted nutrient profiling to assess the nutrient density of plant-based and dairy yogurt products. This profiling provides a holistic approach, taking into consideration multiple nutrients both that are desirable and those that should be limited. In this study, we utilized the Nutrient Rich Foods (NRF) Index, a comprehensive food guidance system that assigns a score to an individual food based on its nutrient density (22). While several other models have been used to assess the nutrient density of foods, the NRF was recently applied to plant-based milk (23), providing an opportunity to compare plant-based products across product categories. The NRF Index is a nutrient profiling method, and its results can inform the food industry on how products can be reformulated (24). In this regard, the plant-based industry can evaluate how the nutrient density of different plant-based yogurts compares to dairy yogurts.

One way the food industry has attempted to address nutrient deficits is through the fortification of vitamins and minerals. Studies have suggested that fortification can improve the nutrient density of plant-based yogurts (15, 25). While this strategy can be effective at increasing the nutrient content, there is also a growing trend of products with a simple ingredient list (13, 26, 27). This becomes challenging within the plant-based category because additional ingredients are needed to provide functional and sensorial properties that mimic conventional animal products (28). This study will contribute to the growing body of research on the inclusion of gums and starches and the fortification of vitamins and minerals in plant-based yogurts by examining the frequency of these additives based on ingredient lists.

As the plant-based yogurt industry continues to launch new products, the nutritional differences between the variety of plant-based and dairy yogurts must be quantified and understood. The objective of this study is to compare the macronutrient and micronutrient profiles of commercially available plant-based and dairy yogurts in the United States using Mintel Global New Products Database, a market research database. Additionally, the NRF Index is used to compare the overall nutrient density of dairy and plant-based yogurts. This information will help the food industry identify areas of opportunity in the nutritional composition of plant-based yogurts to formulate nutritionally dense products.

## 2. Methods

Mintel Global New Products Database (GNPD) was used to generate a database of dairy and plant-based yogurts. From henceforth, this paper will refer to yogurt made from cow milk as “dairy.” The database contained refrigerated yogurts, both flavored and unflavored, launched in the United States between January 2016 and January 2021.

### 2.1. Yogurt database

**Figure 1:**
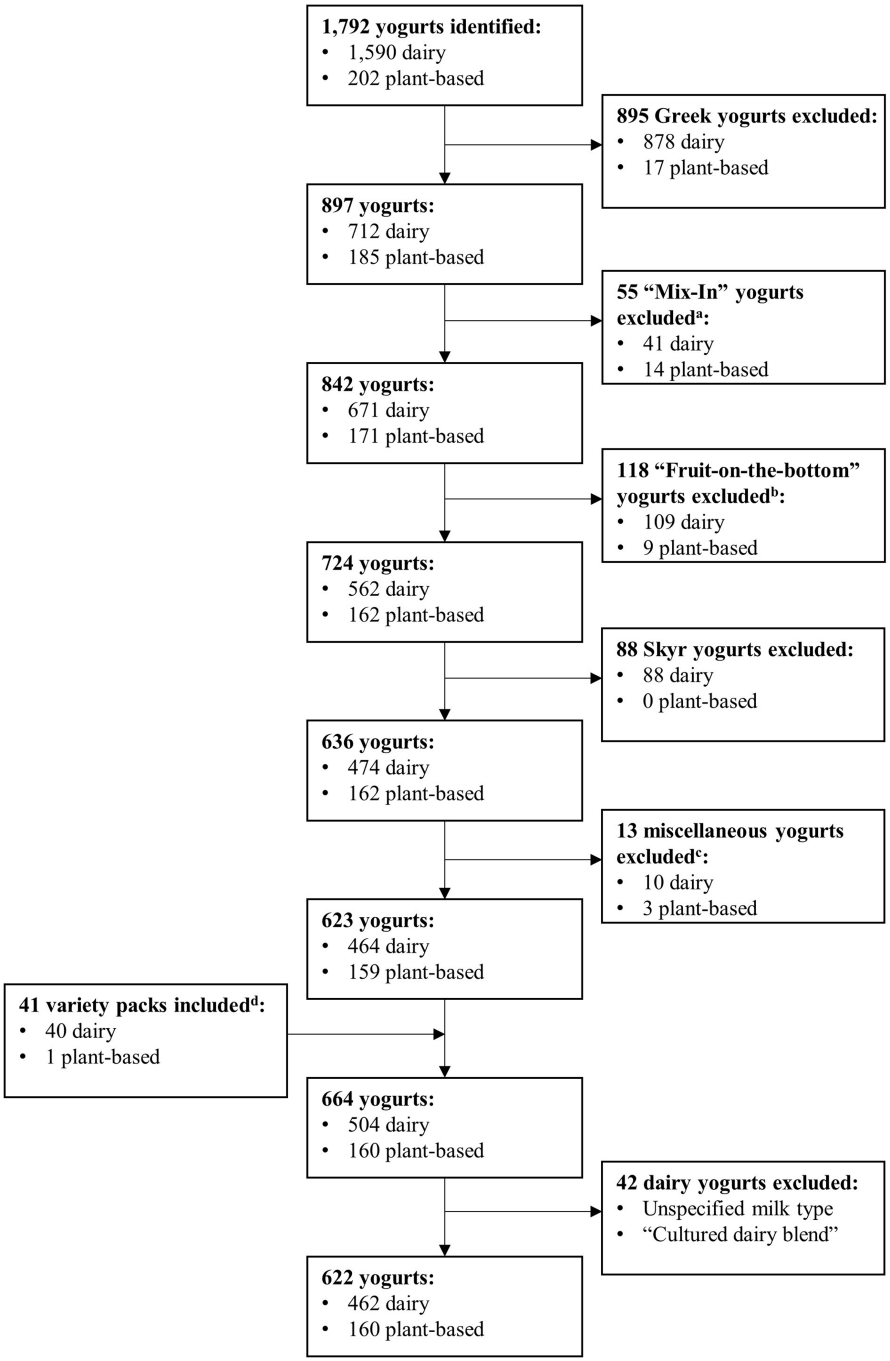
Flow diagram visually depicting the creation of the final database showing step by step inclusion and exclusion criteria. ^a^Product names and/or descriptions that included “mix,” “topping,” “crunch,” “crisp,” “streusel,” “piece,” “slices,” or “granola.” ^b^Products with ingredient layers or described as “on the bottom.” ^c^Kefir and products with missing nutrition facts labels. ^d^Packages with individual products combined in a secondary packaging (variety packs) were considered separate products.

The final database was determined based on the inclusion and exclusion criteria (Figure 1). The database yielded 622 regular-style flavored and unflavored yogurts: 462 dairy and 160 plant-based. Yogurts were categorized according to their primary ingredient, henceforth referred to as the “base.” This was achieved through a review of the ingredient list and a supplemental web search of the product descriptions and images. Bases for dairy yogurt included full-fat (*n* = 159), low-fat (*n* = 288), and nonfat (*n* = 15). Low-fat and nonfat yogurts were consolidated into one base type for statistical analysis due to the Food and Drug Administration’s (FDA) elimination of separate standards of identity for low-fat and nonfat yogurt in 2021 (29). Bases for plant-based yogurt included coconut (*n* = 61), almond (*n* = 44), cashew (*n* = 30), oat (*n* = 15), soybean (*n* = 4), coconut and cashew blend (*n* = 4), and flaxseed (*n* = 2). Due to their small sample size the following yogurt bases were underpowered to analyze nutrient differences: soybean, coconut and cashew blend, and flaxseed yogurts. As a result, these products were excluded from statistical analysis.

Relevant to the present analyses, information in the final database included product, company name, description, serving size (g), ingredient list, and nutritional content. In the U.S., the Nutrition Facts Label must include the following macronutrients and micronutrients: calories, total fat, saturated fat, trans fat, cholesterol, sodium, total carbohydrate, dietary fiber, total sugars, added sugars, protein, vitamin D, calcium, iron, and potassium (30). Nutritional data in the final database thus included energy (kcal), total fat (g), saturated fat (g), carbohydrate (g), fiber (g), protein (g), total sugar (g), sodium (mg), vitamin D (mcg), calcium (mg), iron (mg), potassium (mg), and vitamin B12 (mcg). Nutrient content reported as <0.1 g or < 5 mg was replaced by 0.1 g and 5 mg in the database, respectively. To account for variable serving sizes, nutrient content for all products was reported in grams, milligrams, or micrograms per 100 grams.

During the product search timeframe, the FDA approved changes to the nutrition facts and daily recommended values, which had important implications in the present analysis. In 2016, the FDA updated the Nutrition Facts label (30). Of relevance to the current analyses, these new requirements included updates to reference values used in the declaration of percent Daily Values (DV) of some nutrients (i.e., fat, total carbohydrate, dietary fiber, sodium, potassium, calcium, and vitamin D) and required declarations of micronutrient amounts (i.e., vitamin D, calcium, iron, and potassium). The first phase of modifications was required on January 1, 2020. Thus, the new Nutritional Facts label updates impacted several aspects of this study’s database. See Supplementary Table S1 for description and details on products with new and old nutrition labels and management of nutritional information for the final product database. As a result of these changes that occurred during the timeframe of the product search, some yogurts may be missing one or more nutrients selected for analysis in the present study. Products identified in Mintel GNPD as not reporting one or more nutrients were included in the final database. A researcher performed a manual search to verify nutrients were not reported.

### 2.2. Nutrient profiling

The Nutrient Rich Foods (NRF) Index is a validated comprehensive food guidance system that assigns a score to an individual food based on its nutrient content in relation to calories (31, 32). The NRF Index’s algorithm is based on a positive sub-score for nutrients to encourage (qualifying) and a negative sub-score for nutrients to limit (limiting) (22, 24). The NRF score of an individual food is calculated by the subtraction of the negative sub-score by the positive sub-score. A higher NRF score indicates a higher nutrient density. Previous studies have used different versions of the NRF Index (23, 31, 33, 34), but it has been recommended that for each model the selection of qualifying nutrients be based on nutrients that are beneficial to health (i.e., mainly vitamins and minerals), and limiting nutrients be based on fats, sugars, and sodium (22). In this study, our NRF6.3 model was composed of a positive sub-score for six qualifying nutrients and a negative sub-score for three limiting nutrients. The qualifying nutrients and their reference amounts include protein (50 g), fiber (28 g), calcium (1300 mg), iron (18 mg), potassium (4700 mg), and vitamin D (20 mcg). The limiting nutrients and their reference amounts include saturated fat (20 g), total sugar (50 g) in accordance with previous NRF models (33, 34), and sodium (2300 mg). Each nutrient was expressed as a percentage (%DV) calculated per 100 kcal, where i = qualifying nutrients and j = limiting nutrients (Eq. 1).


(1) $$\begin{array}{l} \mathrm{NRF}6.3=\left(\overset{6}{\underset{i=1}{\sum }}\frac{\mathrm{Nutrient}\;\mathrm{per}\;100\mathrm{kcal}}{\mathrm{Daily Value}\;i\;}*100\right)\\ \;\;\;\;\;\;\;\;\;\;\;\;\;\;\;\;\;\;\;\;\;\;\;-\left(\overset{3}{\underset{j=1}{\sum }}\frac{\mathrm{Nutrient}\;\mathrm{per}\;100\mathrm{kcal}}{\mathrm{Daily Value}\;j\;}*100\right) \end{array}$$

### 2.3. Additive ingredients

To identify the use of additive ingredients, specifically, gums, starches, vitamins, and minerals, ingredient lists were examined for all products in the final product database. The frequency of each ingredient used in each base was recorded.

### 2.4. Statistical analysis

Separate one-way analysis of variance (ANOVA) models were used to compare each nutrient variable across the different yogurt bases. Tukey’s honestly significant difference (HSD) tests were used to test for significant differences in nutrient values among the yogurt bases. For the NRF Index, separate one-way analysis of variance (ANOVA) models were used to compare each score across the different yogurt bases. Tukey’s honestly significant difference (HSD) test was used to test for significant differences in macronutrient and NRF6.3 scores among the yogurt bases, at value of *p* ≤0.001, while *p* ≤ 0.002 was considered significant for micronutrient analysis. Statistical analysis was performed in R (version 4.1.2).

## 3. Results

### 3.1. Macronutrients

**Figure 2:**
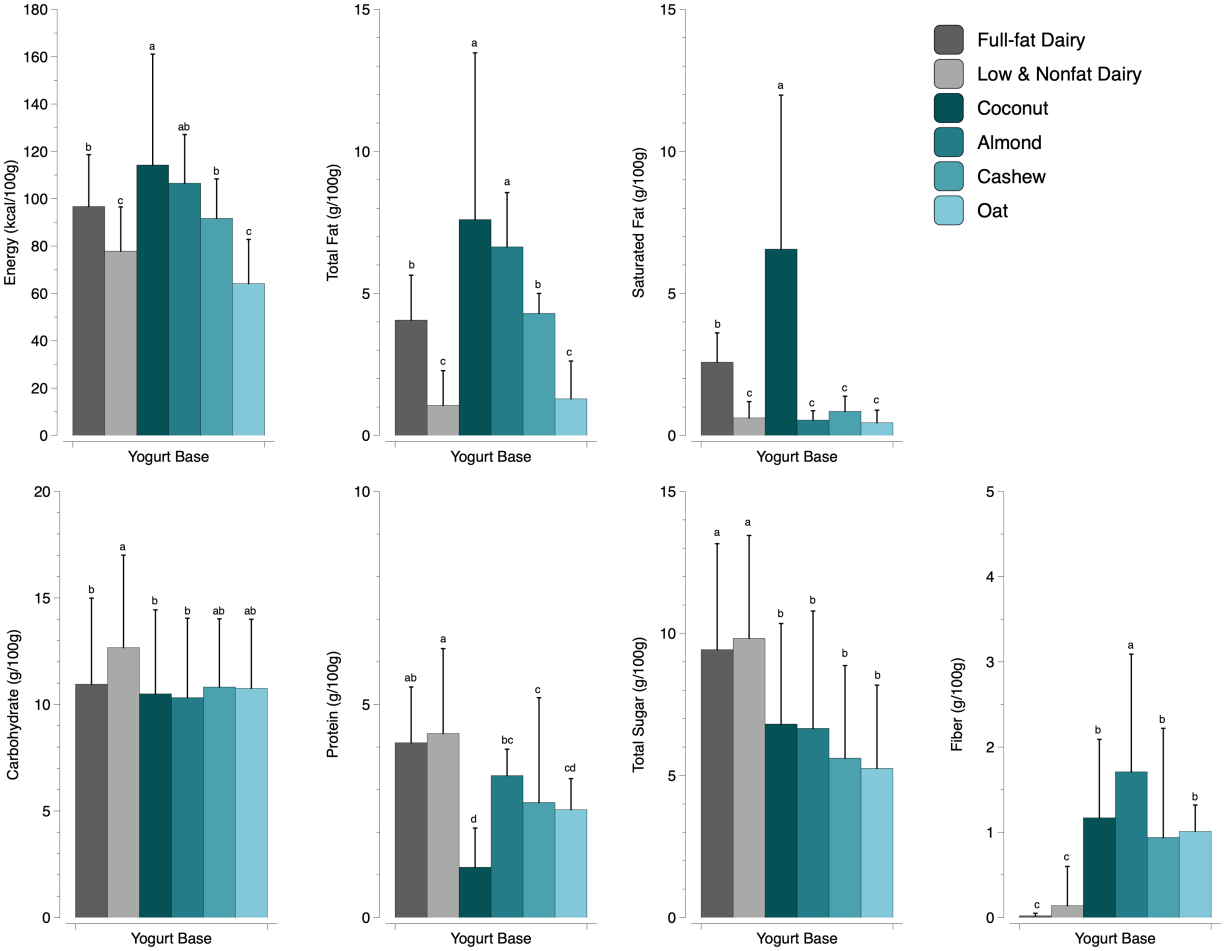
A comparison of macronutrients by yogurt base (*p* ≤ 0.001; values are reported as mean + SD). Different letters within a macronutrient indicate significant differences between yogurt bases. For every macronutrient, we analyzed full-fat dairy (*n* = 159), low and nonfat dairy (*n* = 303), coconut (*n* = 61), almond (*n* = 44), cashew (*n* = 30), and oat (*n* = 15) yogurts.

We analyzed the energy, total fat, saturated fat, carbohydrates, fiber, total sugar, and protein content across 6 different yogurt bases (full-fat dairy, low and nonfat dairy, coconut, almond, cashew, and oat). Significant differences (*p* ≤ 0.001) among yogurt bases were observed for all macronutrients examined (Figure 2 and Supplementary Table S2). A Tukey HSD post-hoc test was conducted to reveal differences between product categories. The energy (kcal/100 g) of the yogurts in the database ranged from oat yogurts’ 64.2 g to coconut yogurts’ 114.2 g. For energy density, full-fat dairy contained more calories per 100 g compared to low and nonfat dairy yogurt. For plant-based yogurts, coconut contained significantly more calories than both dairy groups. Oat yogurt contained significantly fewer calories than full-fat dairy but was not different than the low and nonfat dairy group. Almond and cashew plant-based yogurts were not significantly different from full-fat dairy but were significantly higher than low and nonfat dairy yogurts.

Total fat content was significantly different across product groups. The low and nonfat dairy yogurts and oat yogurts contained the lowest amount of total fat. Coconut and almond yogurts contained significantly more total fat, and full-fat dairy and cashew contained an intermediate amount of total fat. Coconut yogurts contained significantly more saturated fat, followed by full-fat dairy yogurts. There was no difference in saturated fat content among low and nonfat dairy, almond, cashew, and oat yogurts.

For carbohydrate content, we observed the least amount of difference compared to other macronutrients. Low and nonfat yogurts contained significantly more carbohydrates than full-fat dairy. Coconut and almond yogurts were not significantly different from full-fat dairy, and cashew and oat yogurts were not significantly different from either dairy yogurt category. In other words, we observed no significant difference in carbohydrate content among the four categories of plant-based yogurts, which were not different from the full-fat dairy yogurts.

The average protein content for dairy yogurts was roughly 4.2 g per 100 g. There was no difference in protein content between full-fat and low and nonfat dairy yogurts. In comparing plant-based yogurts to dairy yogurts, almond yogurts were found to have a similar protein content as full-fat dairy yogurts but were significantly less than low and nonfat dairy. All other plant-based yogurts contained significantly less protein, with coconut containing significantly less protein than all other products (approximately 1.2 g per 100 g).

The average total sugar content across all product groups ranged from approximately 5 g to almost 10 g per 100 g. There was no difference in sugar content among plant-based products (5.3 g to 6.8 g per 100 g), but they contained significantly less sugar compared to both dairy yogurt groups. There was no significant difference in sugar content among dairy yogurts (9.4g per 100g for full-fat and 9.3g per 100g for low and nonfat).

Almond yogurts contained the highest amount of fiber, which was significantly different from all other yogurt bases. Dairy yogurts contained the least amount of fiber, which was significantly different from all plant-based yogurts (less than 1.2 g per 100 g). Coconut, cashew, and oat yogurts contained intermediate fiber values.

### 3.2. Micronutrients

**Figure 3:**
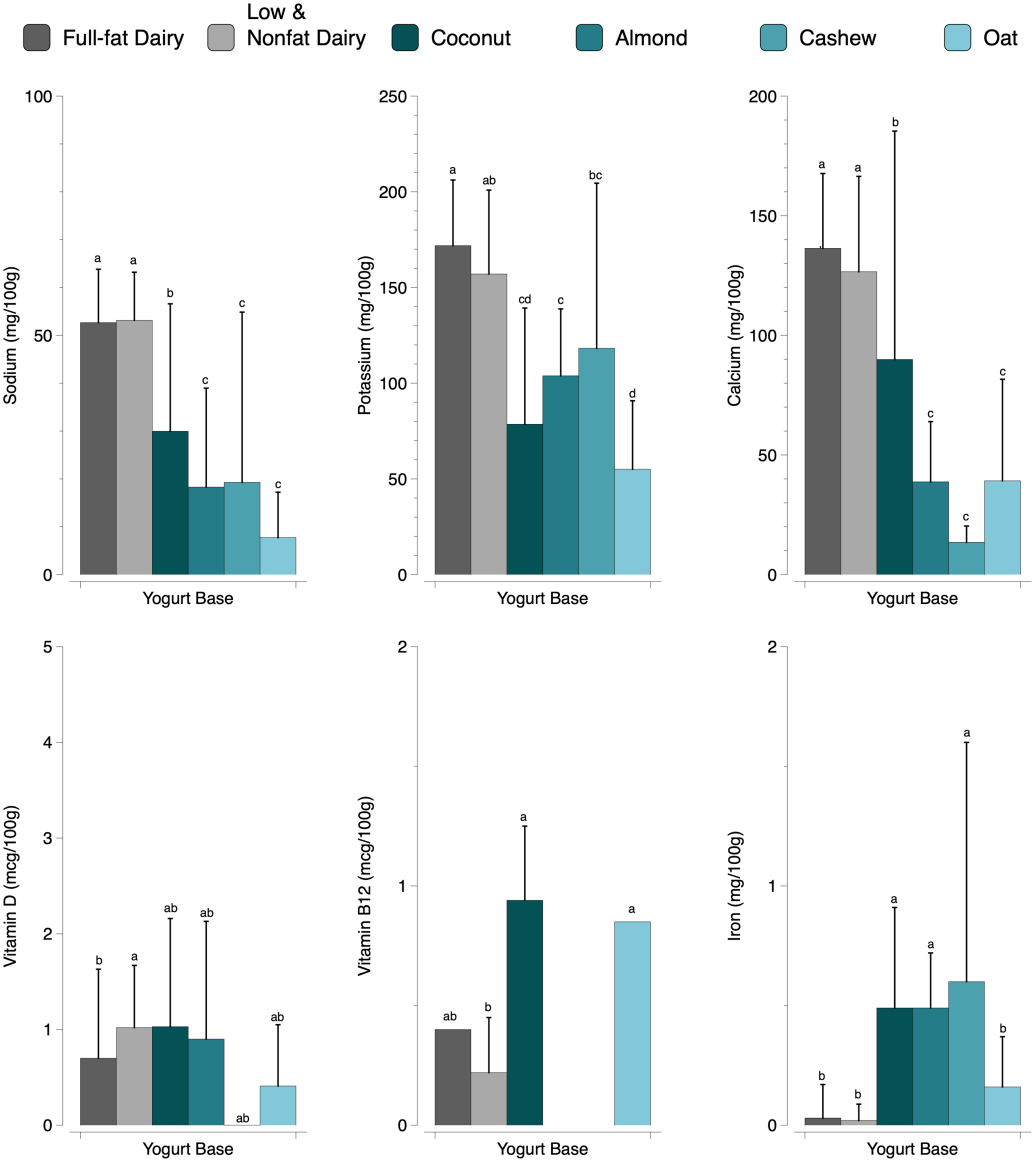
A comparison of micronutrients by yogurt base (*p* ≤ 0.002; values are reported as mean + SD). Different letters within a micronutrient indicate significant differences between yogurt bases. Some products did not report all micronutrients. The following bases (full-fat dairy, low and nonfat dairy, coconut, almond, cashew, and oat, respectively) reported sodium (159, 303, 61, 44, 30, 15); potassium (94, 213, 31, 27, 11, 15); calcium (159, 303, 60, 44, 30, 15); vitamin D (79, 258, 36, 36, 4, 13); vitamin B12 (1, 5, 25, 0, 0, 4); and iron (159, 303, 60, 44, 30, 14).

Analysis of variance was conducted for the following micronutrients: sodium, potassium, calcium, vitamin D, vitamin B12, and iron across 6 yogurt bases (full-fat dairy, low and nonfat dairy, coconut, almond, cashew, and oat). It is important to note that potassium and vitamin D were not required to report on the Nutrition Facts Label before the FDA’s label update. Additionally, vitamin B12 is currently not required to report. Significant differences (*p* ≤ 0.002) among yogurt bases were observed for all micronutrients examined (Figure 3 and Supplementary Table S1). There were significant differences in the sodium content across yogurt bases. Full-fat (52 mg per 100 g) and low and nonfat dairy yogurts (53 mg per 100 g) contained significantly more sodium than all plant-based yogurts. Almond, cashew, and oat yogurts contained significantly less sodium than coconut yogurts. Coconut yogurts contained sodium levels significantly different than all other product categories. Full-fat and low and nonfat dairy yogurts contained significantly more potassium, except for cashew, which was not significantly different from low and nonfat dairy yogurts. Coconut, almond, and oat yogurts contained significantly less potassium. Both dairy yogurt bases contained no difference in the amount of calcium, which was significantly higher than plant-based yogurts. Coconut contained the most amount of calcium among the plant-based yogurts, which was significantly higher than almond, cashew, and oat. There were significant differences in vitamin D content between full-fat and low and nonfat dairy yogurts; however, there were no significant differences between plant-based yogurts. Only 35 yogurts reported vitamin B12 content; these yogurts included full-fat dairy, low and nonfat dairy, oat, and coconut. No products within the almond or cashew groups reported vitamin B12 content. Coconut contained the highest amount of vitamin B12 but was not statistically different from full-fat dairy or oat yogurt. Low and nonfat dairy contained the least amount of vitamin B12 but was not statistically different from full-fat dairy yogurt. Coconut, almond, and cashew yogurts contained significantly more iron than dairy yogurts and oat yogurts.

### 3.3. Nutrient profiling of dairy and plant-based yogurts

**Figure 4:**
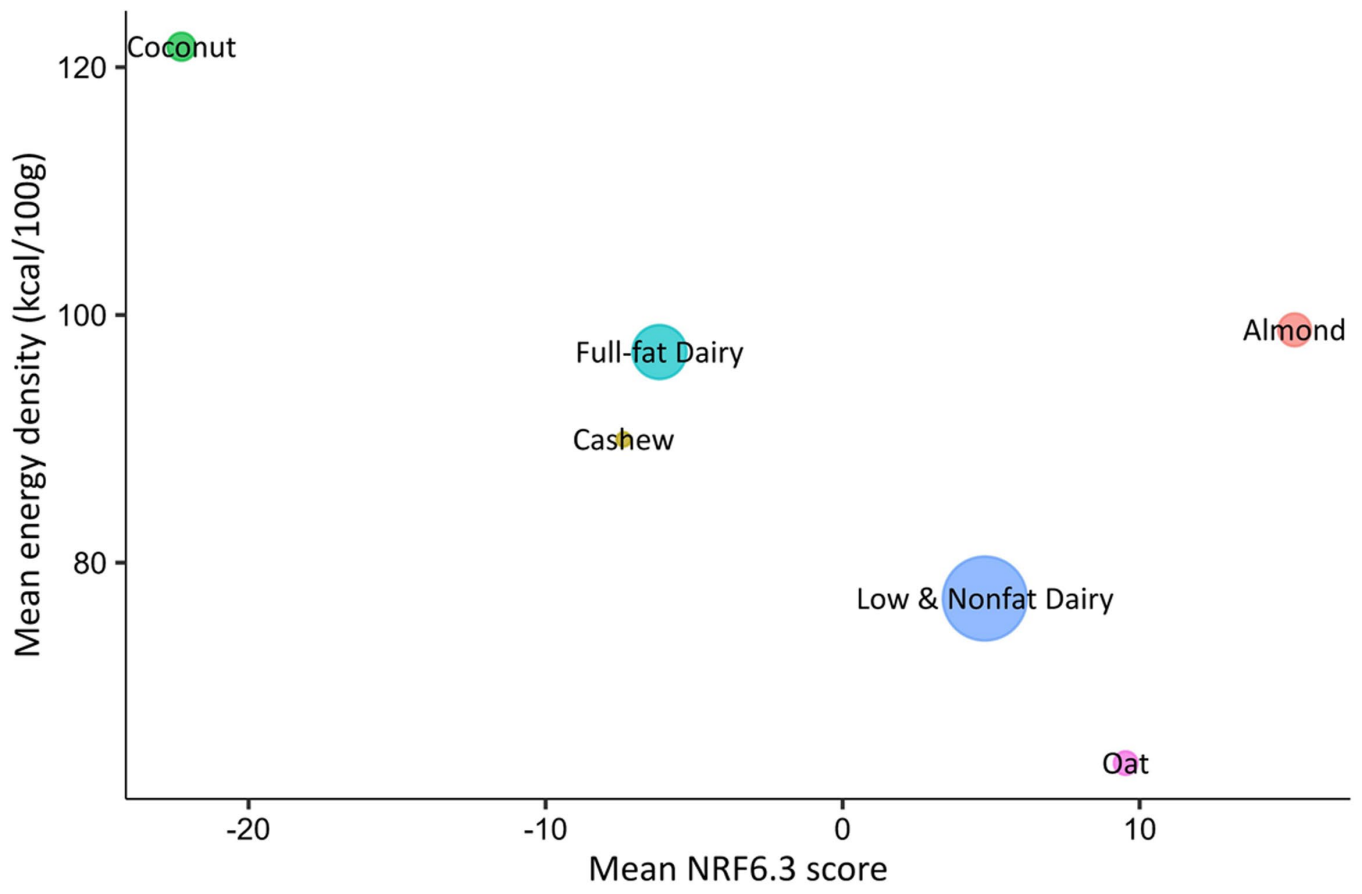
Mean NRF 6.3 scores for each yogurt base type shown in relation to energy density (kcal/100 g). Higher NRF6.3 scores indicate higher nutrient density per 100 kcal. The size of the circle corresponds to the relative number of samples per yogurt base.

For our NRF6.3 model, the following nutrients had to be reported on the product’s label to calculate the NRF score: protein, fiber, calcium, iron, potassium, vitamin D, saturated fat, total sugar, and sodium. Of the 612 yogurts included in this study’s final database, 275 were removed from the NRF model calculations because products did not report one or more nutrient values required for the NRF calculation (See Table 1 for the number of products in each yogurt base). The remaining 337 yogurts were assigned an average score based on their nutrient density (Table 1). NRF6.3 scores ranged from −22.26 to 15.21, and from highest to lowest were almond, oat, dairy low and nonfat, dairy full-fat, cashew, and coconut. A significant difference (*p* ≤ 0.001) was observed for the NRF scores across yogurt bases. Almond yogurts had a significantly higher score than all other yogurts except for oat yogurts. Coconut yogurts had a significantly lower score than all other yogurts except cashew yogurts. Mean NRF6.3 scores in relation to the mean energy density of each yogurt base were mapped (Figure 4). Coconut yogurts had the highest energy density and lowest NRF6.3 score. Oat yogurts had the lowest energy density and the second highest NRF6.3 score.

**Table 1:** A comparison of NRF6.3 scores by yogurt base (*p* ≤ 0.001; values are reported as mean ± SD).

Base	n	Mean ± SD
Almond	27	15.21 ± 14.50^a^
Oat	13	9.95 ± 19.75^ab^
Dairy Low & Nonfat	196	4.79 ± 14.86^b^
Dairy Full-Fat	78	−6.17 ± 10.55^c^
Cashew	4	−7.37 ± 8.48^bcd^
Coconut	19	−22.26 ± 22.00^d^

Different letters within the column indicate significant differences between yogurt bases.

### 3.4. Additive ingredients in dairy and plant-based yogurts

Examining ingredient statements revealed a variety of gums and starches used in this study’s dairy and plant-based yogurts. The frequency of the gum and starch is reported in each product base (Table 2). Yogurts may have contained a combination of multiple gums and starches. Approximately 88.7% of plant-based yogurts and 64.9% of dairy yogurts contained at least one gum. Alternatively, only 17 of the 150 (11.3%) plant-based yogurts did not contain any gums; these yogurts were all coconut yogurts except for one almond yogurt. The most used gum across all yogurts was pectin, present in 308 of the 612 yogurts (50.3%). 67 (14.5%) dairy yogurts contained carrageenan, but this gum was absent in all plant-based yogurts. Corn starch was present in 199 (32.5%) of all yogurts. Both potato and rice starch were used in some plant-based yogurts but were absent in dairy yogurts. Tapioca/cassava starch was used most often in plant-based yogurts, and a majority of cashew and almond yogurts contained this starch.

**Table 2:** Gums and starches present in dairy and plant-based yogurts.

Function	Ingredient	Dairy n = 462		Plant-Based n = 150			
		Full-Fat (n = 159)	Low & Nonfat (n = 303)	Coconut (n = 61)	Almond (n = 44)	Cashew (n = 30)	Oat (n = 15)
Gums	Guar gum	0	5	0	1	4	1
	Locust bean gum	41	22	26	23	26	4
	Xanthan gum	0	29	3	14	0	0
	Pectin	87	139	37	21	17	7
	Agar	0	53	4	17	3	5
	Carrageenan	1	66	0	0	0	0
Starches	Tapioca/Cassava	10	1	9	19	26	3
	Corn	29	150	4	5	7	4
	Potato	0	0	3	0	0	2
	Rice	0	0	20	0	5	0

Additionally, a variety of vitamins and minerals were additive ingredients present in this study’s dairy and plant-based yogurts (Table 3). The addition of calcium salts was more common in plant-based yogurts than dairy yogurts, with 36.7% of plant-based yogurts fortified with calcium while 22.9% of dairy yogurts were enriched with calcium. Calcium citrate and tricalcium/calcium phosphate were most used for both dairy and plant-based yogurts. Vitamin B12 was not present in any dairy yogurts but was used in 41.0% of coconut yogurts. 234 (50.6%) dairy yogurts were enriched with vitamin D3. Vitamin D2 was the most used form of vitamin D in plant-based yogurts. Vitamin E was present in only 3 (5%) coconut yogurts and 4 (1%) low and nonfat yogurts.

**Table 3:** Vitamins and minerals present in the ingredient statement for dairy and plant-based yogurts.

Function	Ingredient	Dairy n = 462		Plant-Based n = 150			
		Full-Fat (n = 159)	Low& Nonfat (n = 303)	Coconut (n = 61)	Almond (n = 44)	Cashew (n = 30)	Oat (n = 15)
Vitamins & Minerals^a^	Calcium citrate	0	4	16	11	0	4
	Calcium/Tricalcium phosphate	1	32	14	8	0	1
	Calcium carbonate	0	0	0	0	0	1
	“Milk calcium”	10	22	0	0	0	0
	Calcium lactate	0	37	0	0	0	0
	Dipotassium phosphate	0	0	9	0	0	0
	Vitamin B12	0	0	25	0	0	4
	Vitamin D	14	8	0	0	0	0
	Vitamin D3	23	211	0	0	0	0
	Vitamin D2	0	0	25	8	0	4
	Vitamin E	0	4	3	0	0	0

a No ingredient lists contained vitamin B2 or vitamin C.

## 4. Discussion

Our findings are consistent with prior work, which demonstrates significant nutritional differences between dairy and plant-based yogurts (15, 17, 18). Despite the growing popularity of plant-based dairy alternatives, the plant-based yogurt category contains variable nutritional compositions in comparison to dairy yogurt. There is an opportunity for the plant-based industry to formulate yogurts that are more nutritionally similar to dairy yogurts. We observed that these nutritional differences in plant-based yogurts are partly due to the use of a variety of ingredients, which help to deliver desirable sensory and textural properties.

### 4.1. Macronutrients

Six studies and one review have previously examined or analyzed the nutritional properties of plant-based yogurts (15–21). In three of the five studies (15, 16, 19–21) that examined nutritional differences between plant-based yogurt bases, coconut yogurts contained the highest number of calories (15, 16, 21). Our results are in line with Clegg and colleagues (15), as coconut yogurts were determined to contain significantly more calories than dairy yogurts.

Previous studies comparing the nutritional composition of dairy and plant-based yogurt consolidated full-fat, low, and nonfat dairy yogurt into one dairy category. As a result, this limited the understanding of how plant-based yogurts compare with dairy yogurts. The present study separated full-fat from low and nonfat dairy yogurts, which resulted in the identification of significant differences in the fat content. For example, coconut yogurts contained significantly higher fat and saturated fat levels than both full-fat and low and nonfat yogurts. On the other hand, low and nonfat yogurts were most similar in saturated fat levels to almond, cashew, and oat yogurts. Coconut yogurts contained significantly higher fat and saturated fat levels than both full-fat and low and nonfat yogurts. This study demonstrates that combining dairy products with varying fat levels into a single category increases the variability within the category and could mask nutritional differences when comparing them to plant-based alternatives (15, 17–19).

In terms of carbohydrates, low and non-fat dairy yogurts contained the highest carbohydrate levels but were not significantly different from cashew and oat yogurts. This contrasts with a previous study, which reported coconut yogurt to have a significantly higher amount of carbohydrates than dairy yogurt (15). Here, almond yogurts contained significantly higher fiber levels than all other yogurts, which supports findings from a previous study (16). Similar to other studies, dairy yogurt fiber levels were significantly lower than plant-based yogurts (18, 19).

Total sugar in all plant-based yogurts was found to be significantly less than full-fat and low and nonfat dairy yogurts. In comparison to previous studies, total sugar results were variable across yogurt products. For studies that compared all dairy yogurts to all plant-based yogurts, the two groups were significantly different in one study (19) which contrasts with two other studies that reported no difference (17, 18). Clegg and colleagues (15) differentiated between plant-based yogurts and found nut-based (i.e., cashew or almond) yogurts to have significantly less total sugar than dairy, coconut, and soy yogurts. The sweetness of plant-based yogurt may provide an important role in masking unpleasant sensory attributes of plant proteins (35). Ingredients such as fruit purees, syrups, and sweeteners may be added (36) to increase the sensorial acceptance of commercial plant-based yogurts. Interestingly, there was no difference in sugar content among plant-based yogurts nor among dairy yogurts. The average sugar content of all plant-based yogurts was approximately 6 g per 100 g while the average sugar content for dairy yogurts was approximately 9.5 g per 100 g. Therefore, plant-based products may provide advantages over dairy yogurts when considering the sugar content.

All studies that compared plant-based yogurts to dairy yogurt found protein levels to be significantly different (15, 17–19). Dairy yogurts contained the highest protein content, except in one review that reported the average protein in soy yogurts to be higher (21). In the present study, low and nonfat dairy yogurts contained significantly higher protein levels than all other yogurts. Additionally, plant-based proteins do not meet the definition of a complete protein because they are missing essential amino acids. To address this limitation, one strategy is to create hybrid or blended products that combine dairy and plant protein. The incorporation of animal protein would help to provide all essential amino acids. However, no products in this study met this definition of a hybrid product. Moreover, there are additional benefits of developing hybrid products, including the positive impact of providing a desirable sensory appeal. Hybrid products may reduce the barriers for reluctant consumers to adopt a more sustainable diet. Indeed, a recent study reported that a blended dairy yogurt with a 25% replacement of plant-based yogurt reported no difference in liking compared to 100% dairy yogurt (37). This suggests partial replacement can help consumers shift to more sustainable options. Another opportunity to improve the protein content is a combination or blend of two or more different plant-based proteins. Four products in this study’s database contained a combination of two plant-protein bases: coconut and cashew. While not included in statistical analysis, these products contained an average protein value of 1.8 g per 100 g, which was less than all other plant-based yogurts except for coconut yogurt (1.2 g per 100 g). There are also additional strategies that could be explored for improving the protein content of plant-based yogurts, such as the utilization of pulses (21) or legumes (16).

It is evident that the previous seven studies, all published from 2020 onward, represent a growing scientific interest in the nutritional properties of plant-based yogurts. Bases included in these studies were coconut (15, 16, 19–21), almond (16, 20, 21), cashew (16, 20, 21), oat (16, 21), soy (15, 16, 20, 21), hemp (20, 21), pea (16, 21), lupin (21), and flaxseed (21). Some studies also combined bases into one category (15, 19) or categorized blends of bases (16). This diversity of bases demonstrates that a blanket statement regarding plant-based yogurts’ nutrition cannot be made. The present study underscores the importance of segmenting plant-based yogurts according to their primary ingredient. Additionally, as consumers continue to adopt a plant-based diet, it is important that these distinctions are made to recognize that not all plant-based yogurts are nutritionally equal.

### 4.2. Micronutrients and fortification of plant-based yogurts

Consumers may adopt a plant-based diet for various reasons, but perceived healthfulness has been identified as an important driver (13, 38). In addition to considering macronutrient content and energy density, it is important to compare the micronutrient content of plant-based yogurt. For example, consumers may consider dairy yogurt to be a source of some vitamins and minerals, such as calcium and vitamin B12. Results from our study differ from previous reports for some nutrients, which may be a result of the base categories. In studies that combined all plant-based yogurt bases into one category and compared to dairy yogurts (17–19), sodium content was not significantly different. In agreement with Clegg and colleagues (15), our results found significant differences in the sodium content between dairy and the different plant-based yogurt bases. However, our results indicate that both full-fat and low and nonfat dairy yogurts contain significantly higher sodium levels than plant-based yogurts. This finding contradicts findings by Clegg and colleagues (15) which reported plant-based yogurts to contain significantly more sodium than dairy yogurts.

As described above, preliminary data suggests that a diet that reduces or removes dairy may create a risk of inadequate consumption of micronutrients such as calcium, vitamin D, potassium, and vitamin B12 (25, 39). Thus, it has been recommended that plant-based dairy substitutes be fortified with micronutrients to compensate for this deficiency (25). In this section, we discuss the vitamin and mineral content alongside the number of products that enhanced their content with fortification.

Calcium levels in plant-based yogurts were significantly lower than in dairy yogurts. In our database, 36.7% of plant-based yogurts’ formulations contained a calcium salt. This suggests some of the products would provide equivalent amounts of calcium, yet on average all plant-based yogurts did not provide equivalent amounts of calcium. As cow’s milk is considered a food rich in calcium (14), fortification of plant-based yogurts is an opportunity for calcium content improvement. In contrast with Clegg and colleagues (15), vitamin D was significantly different amongst the yogurt bases. In this study, 24.7% of plant-based and 56.3% of dairy yogurts contained some form of vitamin D as an additive. Vitamin D fortification, like calcium, is an opportunity for plant-based yogurts to improve from a nutritional standpoint.

In the U.S., potassium is a micronutrient required to be listed on the Nutrition Facts Label (30). However, here we present the first study, both in the U.S. and internationally, to analyze and compare potassium levels in plant-based and dairy yogurts. Non-traditional diets with novel plant-based substitutes did not meet daily potassium requirements (39), so consumers who rely on plant-based yogurts to fulfill this need may find their diets lacking. Full-fat dairy yogurts and low and nonfat dairy yogurts contained an average of 171.9 mg and 157.0 mg of potassium per 100 g, respectively. Future plant-based yogurt nutritional studies should thus include potassium. This is especially important because all plant-based yogurts had lower levels of potassium than dairy yogurts. Fortification of potassium is an opportunity to increase the potassium levels in plant-based yogurts, especially since only 9 plant-based yogurts (all coconut) were fortified with dipotassium phosphate.

Animal-based foods provide sufficient amounts of vitamin B12, whereas fortification is required to incorporate vitamin B12 to plant-based foods. Dairy yogurt is considered a good source of vitamin B12 (e.g., 0.61 mcg of vitamin B12 per 100 g of nonfat yogurt) (14). However, only 29 (19.3%) of this study’s 150 plant-based yogurts were formulated with vitamin B12. Based on our micronutrient analysis, the coconut yogurts that contained vitamin B12 were found to have a significantly higher content compared to low and nonfat yogurts. Craig and Brothers (16) reported that 21.7% of the studied plant-based yogurts had vitamin B12 fortification levels that reached at least 10% DV (DV is 2.4 mcg (40)). These yogurts included coconut, coconut with another ingredient, oat, and a legume-blend. Clegg and colleagues (15) also reported on vitamin B12 content in coconut (0.38 mcg per 100 g) and soy (0.37 mcg per 100 g) yogurts. Consumers reducing dairy products from their diet may need to supplement with vitamin B12 or select plant-based dairy alternatives that include the fortification of vitamin B12. This difference highlights the importance and opportunity to improve the nutritional profile of these yogurts. While this study did not correlate the frequency of additive vitamins and minerals to micronutrient levels, further research can establish the effects of additives on micronutrient properties. Additionally, the bioavailability of plant-based ingredients is variable and is generally lower than animal-based ingredients (28). More work is needed to understand the bioavailability of these ingredients in these food matrices.

### 4.3. Application of the NRF index to dairy and plant-based yogurts

The NRF Index has previously been used to identify dairy yogurt as a highly nutrient-dense food in comparison to popular American snacks (33). However, the NRF Index has not yet been applied to plant-based yogurts. The NRF Index provides an integrative assessment of the nutrient density, expanding on the individual nutrient analysis. It is important to note the high standard deviations observed in the present study for all the NRF6.3 scores for each yogurt base, indicating high variability in the nutrient density for both dairy and plant-based yogurt bases. The NRF6.3 Index was based on a positive sub-score for protein, fiber, calcium, iron, potassium, and vitamin D; and a negative sub-score for total sugar, sodium, and saturated fat. A higher NRF score indicates a higher nutrient density. In our study, almond and oat yogurts scored higher than full-fat and low and nonfat dairy yogurts. Therefore, almond and oat yogurts are more nutritionally dense than dairy yogurts. Based on our macronutrient and micronutrient analysis, almond and oat yogurts’ higher NRF scores can be attributed to their low levels of total sugar, sodium, and saturated fat. All three of these macronutrient levels were lower in almond and oat yogurts than dairy yogurts. Regarding sodium and total sugar content, almond and oat yogurts contained significantly lower levels than dairy yogurts. Though cashew yogurts had a lower NRF score than both dairy categories, the scores were not significantly different. This indicates that cashew yogurt has a similar nutrient density to dairy yogurts. Coconut yogurt had the lowest nutrient density, likely due to its high saturated fat content and low protein levels. As almond and oat yogurts are already more nutrient dense than dairy yogurts, further product development can address any sensorial disparities compared to dairy yogurts. Coconut yogurts may need reformulation to first address their distinct nutritional profiles compared to dairy yogurts.

A previous study has used a different nutrient profiling method for plant-based yogurts (19), and a different study used the NRF Index for plant-based milk alternatives (23). Katidi and colleagues (19) utilized the Nutri-Score algorithm to assess the nutrient quality of plant-based yogurts available in Greece. They reported that most plant-based yogurts were given a higher Nutri-Score than their dairy equivalents. Drewnowski (34) utilized the NRF Index to examine the nutrient density of plant-based milk alternatives (23), which is most comparable to this study’s NRF results. Drewnowski’s model (NRF5.3) contained the same limiting nutrients as the present study; however, for qualifying nutrients, it included vitamin A and vitamin B12, which were not used in the present study. In our study and Drewnowski’s study, the order of the NRF scores from highest to lowest was the same: almond, oat, cashew, and coconut.

While this study’s NRF model provides a consistent way to compare the overall nutrient profile, which considers nutrients that should be included and limited in a food product, there are some limitations of this approach in the present study. Prior to the calculation of the NRF6.3 score, 45% of the products in the database were removed due to missing one or more macronutrients and/or micronutrients selected for the model. This was largely due to missing vitamin D and potassium values, as the FDA did not require reporting these micronutrients prior to 2020. Despite this limitation, the present analysis is the first to use the NRF Index for dairy and plant-based yogurts. The NRF Index provides an advantage when comparing the nutritional compositions of products. Rather than comparing individual nutrients of a product to another product, the NRF Index examines the overall nutrient density of a product (9).

Overall, using the NRF Index to analyze the nutritional profiles of yogurts provides valuable insight into the nutrient density of commercially available plant-based yogurts. The NRF6.3 scores provide a holistic view of the nutritional quality of the plant-based products in comparison to conventional yogurt products. This study highlights that some product categories may provide advantages regarding the overall nutrient quality, yet the industry needs to continue to consider individual macronutrients and micronutrients to improve the overall nutrient composition.

### 4.4. Additive ingredients in plant-based yogurts

Sensorial properties have been identified as barriers to plant-based dairy consumption (26). Raw plant materials are known to evoke bitter, beany, astringent, herbaceous tastes, and odors, which can be unappealing to dairy yogurt consumers (36). Specific to plant-based yogurts, the texture was found to have a major effect on product liking (41). For example, mouthfeel at the beginning of mastication was found to have an important effect on the liking of oat yogurts (42). The poor textural profile of plant-based yogurts has been attributed to the lack of globular plant proteins’ ability to mimic/recreate the molecular attributes of casein (26). The food industry and researchers have noted significant challenges in recreating the textural and mouthfeel characteristics of plant-based products (36, 43). Therefore, the addition of thickeners and structural agents (i.e., gums and starches) contributes to the viscosity of plant-based gels (44) and may create a more sensorially acceptable plant-based yogurt product.

Here, we examined the incorporation of thickening agents in both dairy and plant-based yogurts. Gums can be used in plant-based yogurts to prevent syneresis (45) and can be utilized as thickening agents (46). In our study, many plant-based yogurts used gums in their formulations; pectin and locust bean gum were the most popular. Similar to Boeck and colleagues (21) and Craig and Brothers (16), the most used gums in the plant-based yogurts were pectin (54.7%), locust bean gum (52.7%), and agar (19.3%) Like gums, starches can be used in plant-based products for gelling, binding, and stabilizing purposes (47). Similar to Boeck et al. (21), the most commonly used starch was tapioca (38.0%).

Gums and starches are included in plant-based yogurts to increase consumer acceptance by improving the sensory and mouthfeel characteristics (25, 48). However, the use of these ingredients may conflict with the recent and growing consumer trend of a “clean label” defined by a simple ingredient list and minimal processing (13, 26, 27). Overall, almond yogurt contained the second-highest total number of gums and starches in all yogurts, but the highest NRF6.3 score. Almond yogurts are the most popular plant-based yogurt purchased in the U.S. (9), suggesting that consumers may be willing to accept additive ingredients if the product is nutrient dense. Further research is needed to measure how willing consumers are to accept plant-based yogurts with a high nutrient composition but also a high additive count.

## 5. Conclusion

As consumers adopt plant-based diets for health reasons, it is important to examine the extent to which plant-based yogurt alternatives match the nutritional profile of dairy yogurts. Our analysis, based on data from Mintel GNPD for commercial products in the U.S., demonstrated that while plant-based yogurts contain less total sugar, less sodium, and more fiber than dairy yogurts, they contain less protein, calcium, and potassium than dairy yogurts. A nutrient assessment revealed that when considering key nutrients provided by dairy yogurts, oat yogurts were the most similar to low and nonfat dairy yogurts. Further, when considering the nutrient density and the energy density of commercial yogurts, oat and almond appeared to be similar or better compared to dairy yogurts, while cashew yogurts were similar to low and nonfat dairy yogurts. Coconut had the lowest nutrient density and highest energy density. Fortification with vitamins and minerals has the potential to improve the nutritional profile (e.g., calcium and vitamin B12). With the current strategy for plant-based products to be substituted for conventional dairy products, there is a need to consider the desirable nutritional benefits of dairy yogurt, such as protein, vitamin B12, and calcium, and the minimization of nutrients such as total sugar, sodium, and saturated fat.

## Data availability statement

The raw data supporting the conclusions of this article will be made available by the authors, without undue reservation.

## Author contributions

AD’A, AK, and AN contributed to the conception and design of the study, interpretation of the results, and wrote and/or contributed to all sections of the manuscript. AD’A organized the database, performed the statistical analysis, and wrote the first draft of the manuscript. All authors contributed to the article and approved the submitted version.

## Funding

This material is based upon work supported by the National Institute of Food and Agriculture, U.S. Department of Agriculture, the Center for Agriculture, Food and the Environment, and the Department of Food Science at University of Massachusetts Amherst, under project numbers MAS-00529 and MAS-00491. The contents are solely the responsibility of the authors and do not necessarily represent the official views of the USDA or NIFA.

## Conflict of interest

The authors declare that the research was conducted in the absence of any commercial or financial relationships that could be construed as a potential conflict of interest.

## Publisher’s note

All claims expressed in this article are solely those of the authors and do not necessarily represent those of their affiliated organizations, or those of the publisher, the editors and the reviewers. Any product that may be evaluated in this article, or claim that may be made by its manufacturer, is not guaranteed or endorsed by the publisher.
