# Supplementary material for: A comparison of the nutritional profile and nutrient density of commercially available plant-based and dairy yogurts in the United States
Source: Front Nutr. 2023 May 25;10:1195045. doi: 10.3389/fnut.2023.1195045 (PMC10248066; doi:10.3389/fnut.2023.1195045)
Supplement: Supplementary file 1 [file Data_Sheet_1.docx]

Supplementary Material

A Comparison of the Nutritional Profile and Nutrient Density of Commercially Available Plant-Based and Dairy Yogurts in the United States

Astrid E. D’Andrea, Amanda J. Kinchla, Alissa A. Nolden*

*** Correspondence:** Alissa A. Nolden: anolden@umass.edu

# Old and updated nutrition facts label database modifications

The range of dates for this database spanned from 2016 to 2021; therefore, nutrient amount calculations were performed in accordance with the percent DVs established at the time. The following are label changes that affected the database and how these changes were addressed:

- Vitamin A and vitamin C are not required: excluded from this study’s analyses, supplementary data, figures and/or tables
- Vitamin D and potassium in micrograms and milligrams, respectively are required: if a product with the old label contained the percent DV, this was used to calculate the micronutrient amount; if a product with the old label did not contain the percent DV, “NA” was recorded for the micronutrient amount
- Calcium and iron are listed in milligrams in addition to percent DV: products with the old label contained only the percent DV, so this percentage was used to calculate the micronutrient amount

Table S1. Summary of the number of dairy and plant-based products with the old and updated Nutrition Facts label per year (January 2016 – January 2021).

| **Year** | **Old Label** | | **Updated Label** | | **Total** |
| --- | --- | --- | --- | --- | --- |
|  | **Dairy** | **Plant-Based** | **Dairy** | **Plant-Based** |  |
| **2016** | 105 | 20 | 0 | 0 | 125 |
| **2017** | 105 | 28 | 9 | 0 | 142 |
| **2018** | 71 | 17 | 11 | 8 | 107 |
| **2019** | 17 | 27 | 75 | 27 | 146 |
| **2020** | 1 | 0 | 56 | 30 | 87 |
| **2021** | 0 | 0 | 12 | 3 | 15 |

# Macronutrient results

Table S2. A comparison of macronutrients by yogurt base (p ≤ 0.001; values are reported as mean ± SD).

| **Macronutrient** | **Full-Fat Dairy (n = 159)** | **Low & Nonfat Dairy (n = 303)** | **Coconut (n = 61)** | **Almond (n = 44)** | **Cashew (n = 30)** | **Oat (n = 15)** |
| --- | --- | --- | --- | --- | --- | --- |
| Energy (kcal/100g) | 96.74 ± 21.88^b^ | 77.84 ± 18.69^c^ | 114.23 ± 46.86^a^ | 106.56 ± 20.57^ab^ | 91.72 ± 16.64^b^ | 64.18 ± 18.63^c^ |
| Total Fat (g/100g) | 4.06 ± 1.58^b^ | 1.06 ± 1.22^c^ | 7.60 ± 5.87^a^ | 6.64 ± 1.91^a^ | 4.30 ± 0.70^b^ | 1.29 ± 1.33^c^ |
| Saturated Fat (g/100g) | 2.58 ± 1.03^b^ | 0.62 ± 0.57^c^ | 6.56 ± 5.42^a^ | 0.54 ± 0.33^c^ | 0.85 ± 0.53^c^ | 0.45 ± 0.44^c^ |
| Carbohydrates (g/100g) | 10.95 ± 4.04^b^ | 12.67 ± 4.34^a^ | 10.50 ± 3.94^b^ | 10.32 ± 3.73^b^ | 10.82 ± 3.20^ab^ | 10.76 ± 3.24^ab^ |
| Protein (g/100g) | 4.10 ± 1.31^ab^ | 4.32 ± 1.99^a^ | 1.18 ± 0.92^d^ | 3.33 ± 0.62^bc^ | 2.70 ± 2.46^c^ | 2.53 ± 0.73^cd^ |
| Total Sugar (g/100g) | 9.43 ± 3.73^a^ | 9.83 ± 3.62^a^ | 6.81 ± 3.54^b^ | 6.66 ± 4.13^b^ | 5.61 ± 3.26^b^ | 5.25 ± 2.93^b^ |
| Fiber (g/100g) | 0.02 ± 0.03^c^ | 0.14 ± 0.46^c^ | 1.17 ± 0.92^b^ | 1.71 ± 1.38^a^ | 0.94 ± 1.28^b^ | 1.01 ± 0.31^b^ |

Different letters within a row indicate significant differences between yogurt bases.

# Micronutrient results

Table S3. A comparison of micronutrients by yogurt base (p ≤ 0.002; values are reported as mean ± SD).

| **Micronutrient** | | **Full-Fat Dairy** | **Low & Nonfat Dairy** | **Coconut** | **Almond** | **Cashew** | **Oat** |
| --- | --- | --- | --- | --- | --- | --- | --- |
| Sodium  (mg/100g) | n  Mean | 159  52.65 ± 11.16^a^ | 303  53.14 ± 10.05^a^ | 61  29.95 ± 26.64^b^ | 44  18.30 ± 20.66^c^ | 30  19.27 ± 35.58^c^ | 61  7.73 ± 9.49^c^ |
| Potassium (mg/100g) | n  Mean | 94  171.87 ± 34.27^a^ | 213  157.02 ± 43.87^ab^ | 31  78.55 ± 60.69^cd^ | 27  103.87 ± 34.93^c^ | 11  118.27 ± 86.23^bc^ | 15  55.05 ± 35.77^d^ |
| Calcium (mg/100g) | n  Mean | 159  136.37 ± 31.27^a^ | 303  126.56 ± 39.87^a^ | 60  89.91 ± 95.45^b^ | 44  38.79 ± 25.15^c^ | 30  13.49 ± 6.80^c^ | 15  39.19 ± 42.45^c^ |
| Vitamin D (mcg/100g) | n  Mean | 79  0.70 ± 0.93^b^ | 258  1.02 ± 0.65^a^ | 36  1.03 ± 1.13^ab^ | 36  0.90 ± 1.23^ab^ | 4  0.00 ± 0.00^ab^ | 13  0.41 ± 0.64^ab^ |
| Vitamin B12 (mcg/100mg) | n  Mean | 1  0.40 ± 0.00^ab^ | 5  0.22 ± 0.23^b^ | 25  0.94 ± 0.31^a^ | 0  n/a | 0  n/a | 4  0.85 ± 0.00^a^ |
| Iron (mg/100g) | n  Mean | 159  0.03 ± 0.14^b^ | 303  0.02 ± 0.068^b^ | 60  0.49 ± 0.42^a^ | 44  0.49 ± 0.23^a^ | 30  0.60 ± 1.00^a^ | 14  0.16 ± 0.21^b^ |

Different letters within a row indicate significant differences between yogurt bases.
